# Macrophages with Different Polarization Phenotypes Influence Cementoblast Mineralization through Exosomes

**DOI:** 10.1155/2022/4185972

**Published:** 2022-09-16

**Authors:** Yi Zhao, Yiping Huang, Hao Liu, Kuang Tan, Ruoxi Wang, Lingfei Jia, Weiran Li

**Affiliations:** ^1^Department of Orthodontics, Peking University School and Hospital of Stomatology, Beijing 100081, China; ^2^Department of Oral and Maxillofacial Surgery, Peking University School and Hospital of Stomatology, Beijing 100081, China; ^3^Central Laboratory, Peking University School and Hospital of Stomatology, Beijing 100081, China

## Abstract

Root resorption is a common dental challenge that can lead to tooth loosening or even tooth loss. Among the cells involved in root resorption, cementoblasts are responsible for laying down the cementum, while macrophages with different phenotypes have also been shown to have bidirectional effects on root resorption. However, the relationship between macrophages and cementoblasts remains largely unknown. In this study, we examined the effect of macrophages with different polarization phenotypes on the mineralization of cementoblasts. Using the transwell coculture system and a conditioned medium-based coculture system, we found that compared with M0 (unpolarized macrophages), M1-polarized macrophages attenuated cementoblast mineralization, while M2-polarized macrophages enhanced cementoblast mineralization. Furthermore, by extracting M0/M1/M2 macrophage exosomes and examining their effects on the mineralization of cementoblasts, we found that the effects of macrophages on cementoblast mineralization were, at least partially, exerted by exosomes. Moreover, *in vivo* studies also indicated that an increased M1/M2 ratio could suppress cementoblast mineralization and bring about root resorption. During mechanical force-induced orthodontic tooth movement (OTM), root resorption was evident on the compression side of periodontal tissue, and a higher M1/M2 ratio and weaker cementoblast mineralization were observed on the compression side than on the tension side. We also used localized lipopolysaccharide (LPS) injection to increase the M1/M2 ratio around the roots of maxillary molars, where root resorption and decreased cementoblast mineralization were also observed. Furthermore, when we injected the exosomes from M0 and M1- and M2-polarized macrophages into mice, it was observed that the cementoblast mineralization was attenuated in the group injected with M1-polarized macrophage exosomes, while it was augmented in the group injected with M2-polarized macrophage exosomes.

## 1. Introduction

Tooth root resorption is a common clinical challenge in dentistry and can be divided into several types, including internal, external, invasive, pressure, and idiopathic resorption [1]. Among them, external root resorption is the most common. Root resorption can also be classified based on stimulation factors, including orthodontic pressure resorption, periodontal infection resorption, pulpal infection resorption, impacted tooth or tumor pressure resorption, and ankylotic resorption [2]. Orthodontic pressure resorption is the most common side effect during orthodontic treatment.

The cementum is a thin layer on the root surface that serves as an antiresorptive barrier and anchorage for a functional periodontal ligament [3, 4]. Cementoblasts, which are responsible for laying down the cementum, like osteoblasts, express the transcription factors of bone sialoprotein (BSP), osterix (OSX), alkaline phosphatase (ALP), osteocalcin (OCN), and runt-related gene 2 (RUNX2) [3, 5]. Among these, OSX is important in transactivating genes encoding type 1 collagen and upregulating cell differentiation and mineralization [3]. Apart from the primary role of synthesizing the components of cementum, cementoblasts have also been reported to express superoxide dismutase 3, which plays a key role in defending against oxidative stress during cementum maintenance [4, 6]. In addition, cementoblasts are also relevant to osteoclastogenesis, as Huynh et al. proposed that cementoblasts had the capacity to induce osteoclastogenesis, which was strongly promoted by IL-1*β* [7]. Nemoto et al. also demonstrated that cementoblasts expressed functional Toll-like receptors, which were involved in altered gene expression related to cementum formation and upregulation of osteoclastogenesis-related molecules [8].

Macrophages are indispensable immune cells that contribute to both proinflammatory and anti-inflammatory processes and are involved in both tissue destruction and regeneration [9]. Polarized macrophages can be divided into two main groups. M1-polarized macrophages are also called classically activated macrophages. Typical activating stimuli of M1 macrophages are interferon-*γ* (IFN-*γ*) and lipopolysaccharide (LPS)^9^. M2-polarized macrophages are also called alternatively activated macrophages. M2 macrophages can be further classified into M2a macrophages, stimulated by IL-4 or IL-13; M2b macrophages, stimulated by immune complexes in combination with IL-1*β* or LPS; and M2c macrophages, stimulated by IL-10, TGF-*β*, or glucocorticoids [9]. M1-polarized macrophages function in proinflammatory processes and can secrete significant amounts of proinflammatory cytokines, such as IL-1*β*, IL-12, IL-15, IL-18, and TNF-*α* [9]. Conversely, M2-polarized macrophages are critical in the anti-inflammatory and tissue repair phases [9]. Macrophages also play important roles in regulating the metabolic activity of teeth and periodontal tissue. Macrophages have been reported to mediate LPS-induced apoptosis of osteoblasts and periodontal ligament cells through the induction of TNF-*α* [10]. In addition, several lines of evidence have demonstrated that exosomes from M0 (unpolarized macrophages) and M2-polarized macrophages promoted bone repair and regeneration, while exosomes from M1-polarized macrophages inhibited bone repair [11]. In the case of orthodontic tooth movement (OTM), compression of the vascular system caused by local orthodontic load and subsequent ischemia of the periodontium lead to the necrosis and hyalinization of the periodontal ligament and alveolar bone [3]. Subsequently, macrophage-like cells, multinucleated cells, osteoclasts, cementoclasts, and odontoclasts remove the necrotic and hyalinized tissues, and this process causes the side effect of external root resorption [4]. The cellular process of external root resorption is broadly similar to that of bone resorption [4]. He et al. demonstrated that the M1/M2-polarized macrophage ratio was increased and root resorption was exacerbated at the force application period during OTM [12]. After the removal of force, the M1/M2-polarized macrophage ratio was decreased, and root resorption was partially rescued [12]. On that basis, Li et al. conducted *in vitro* experiments to demonstrate that M2-polarized macrophages could enhance the cementoblastic differentiation of periodontal ligament stem cells [13]. Further explorative experiments found that the M1 polarization markers IL-1*β*, IL-6, and TNF-*α* could attenuate the mineralization of cementoblasts, while the M2 polarization marker PPAR*γ* promoted cementoblast mineralization [14–17]. These results all suggested a potential linkage between macrophages, cementoblasts, and root resorption.

Exosome is a type of membrane vesicles that most types of cells can secrete into the extracellular environment [18]. It can transport miRNAs, mRNAs, proteins, and other substances to play many biological roles, especially in intercellular communication [19]. As previous studies have described the different effects of exosomes from macrophages with different polarization phenotypes on bone metabolism [11], it is hypothesized that exosomes may exert similar effects on the metabolism of cementum.

However, the way macrophages and their related molecules affect root resorption, including causing root resorption and promoting its repair, is still unclear. Since no direct evidence has been found in the relationship between macrophages and cementoblast mineralization, whether macrophages with different polarization phenotypes show a similar or different effect on the mineralization of cementoblasts remains unknown. In this study, we aimed to clarify the effect of M0 and M1- and M2-polarized macrophages on cementoblast mineralization and preliminarily explore the underlying mechanisms, in order to provide a better understanding of the cellular mechanism of root resorption and repair.

## 2. Materials and Methods

### 2.1. Cell Culture and Polarization of Macrophages

RAW 264.7 murine monocyte-like cells were purchased from ScienCell (San Diego, CA, USA). The immortalized murine cementoblastic cell line OCCM-30 was generously provided by Dr. Martha J. Somerman (National Institutes of Health, Bethesda, MD) [20]. The cells were cultured in Dulbecco's modified Eagle's medium (DMEM; Gibco, Gaithersburg, MD, USA) with 10% fetal bovine serum (FBS; Gibco, Gaithersburg, MD, USA) supplemented with 1% penicillin-streptomycin solution (Gibco, Gaithersburg, MD, USA). Cells were incubated at 37°C in 5% CO_2_ in humidified air. Assays were performed when the culture reached 75-80% confluence.

For M1 polarization, cells were treated with 2.5 ng/ml IFN-*γ* (Sigma-Aldrich, St. Louis, MO, USA) and 200 ng/ml LPS (Sigma-Aldrich, St. Louis, MO, USA) for 12 h before assays. For M2 polarization, cells were treated with 50 ng/ml IL-4 for 12 h.

### 2.2. Transwell Coculture System

In a transwell coculture system of cementoblasts and macrophages with different polarization phenotypes, cementoblasts were seeded in the upper inserts (0.4 *μ*m pore; Corning, Lowell, MA) and M0/M1/M2 macrophages were seeded and induced in 12-well plates. Then, the upper inserts were placed onto the 12-well plates, wherein M0/M1/M2 were incubated. The 12-well plates were changed every day to provide freshly induced M0/M1/M2. After a 3-day coculture, cementoblasts were collected for RNA and protein extraction.

### 2.3. Conditioned Medium Preparation and Coculture

After a 24 h incubation of M0/M1/M2 with DMEM (without FBS), the medium was collected separately. Then, the supernatant, which was extracted from the medium by centrifugation at 300 × *g* and 2000 × *g* for 10 min, respectively, was mixed with DMEM (with FBS) at a ratio of 1 : 1. The mixtures of M0/M1/M2 supernatant and DMEM (with FBS) were used as the conditioned medium for cementoblasts. During the 3-day incubation of cementoblasts, the conditioned medium was refreshed every day with newly produced supernatant.

### 2.4. Exosome Purification

After a 24 h incubation of M0/M1/M2 with DMEM (without FBS), the medium was collected separately. First, cells were removed by centrifugation at 300 × *g* for 10 min. Then, dead cells were removed by centrifugation at 2000 × *g* for 10 min, followed by cell debris removal by centrifugation at 10,000 × *g* for 30 min. The exosomes were initially collected by ultracentrifugation at 100,000 × *g* for 70 min, washed in Phosphate-Buffered Saline (PBS), and finally collected by ultracentrifugation under the same condition again. The final output of exosomes was stored at −40°C and verified by western blot analysis and electron microscopy. M0/M1/M2 exosomes were added to the culture medium of cementoblasts at a dose of 150 *μ*g/ml every day. After a 3-day incubation, the cementoblasts were subjected to RNA or protein extraction or immunofluorescence staining.

### 2.5. RT-qPCR

RT-qPCR was performed as previously described [21]. Total RNA was extracted from macrophages or cementoblasts using TRIzol reagent, and cDNA was reverse transcribed by a PrimeScript RT Reagent Kit. RT-qPCR was conducted using FastStart Universal SYBR Green Master Mix on a Real-Time PCR Detection System. Glyceraldehyde 3-phosphate dehydrogenase (GAPDH) mRNA served as the internal normalization control. The primer sequences of GAPDH, BSP, OSX, RUNX2, collagen, type I, alpha 1 (COL-1), protein-tyrosine phosphatase-like member A (PTPLA), inducible nitric oxide synthase (iNOS), and Arginase 1 (Arg-1) are listed in Table 1.

### 2.6. Western Blot Analysis

Western blot analysis was performed as previously described [21]. Macrophages or cementoblasts were washed and solubilized with RIPA lysis buffer. Protein concentrations were measured and adjusted to be the same, followed by electrophoresis on a precast gel and then transferred to a polyvinylidene difluoride membrane. After being blocked with 5% BSA, the transferred membranes were incubated at 4°C overnight with anti-iNOS antibody, anti-ALIX antibody, anti-GM130 antibody (Proteintech, Chicago, IL, USA), anti-CD63 antibody, anti-Arg-1 antibody, anti-BSP antibody, anti-COL-1 antibody (Cell Signaling Technology, Danvers, MA, USA), anti-RUNX2 antibody, anti-OSX antibody, or anti-*β*-actin antibody (Abcam, Cambridge, MA, USA) diluted at 1 : 1000. Subsequently, the membranes were incubated with anti-rabbit or anti-mouse secondary antibodies (ZB-2301 and ZB-2305, Zhongshan Golden Bridge Biotechnology, Beijing, China), which were diluted at 1 : 5000 at room temperature for 1 h. The expression of associated proteins was visualized with a chemiluminescence reagent and analyzed using ImageJ software.

### 2.7. Immunofluorescence Staining and Fluorescence Imaging

Immunofluorescence staining was performed as previously described [21]. After a 3-day incubation with exosomes from M0/M1/M2 macrophages, cementoblasts were fixed with 4% paraformaldehyde in PBS, permeabilized with 0.1% Triton X-100, and blocked with 5% goat serum. Then, cementoblasts were incubated with anti-OCN antibody (Proteintech, Chicago, IL, USA) diluted at 1 : 200 at 4°C overnight. After incubating cells with secondary antibodies, nuclei were stained with DAPI, and images were collected using a confocal imaging system.

### 2.8. ALP Staining

A 5-bromo-4-chloro-3-indolyl-phosphate/nitro blue tetrazolium staining kit (NBT/BCIP, CoWin Biotech, Beijing, China) was used as previously described [22]. After a 5-day application of exosomes from M0 and M1- and M2-polarized macrophages, cementoblasts were washed with PBS and fixed in 4% paraformaldehyde for 30 min. After three washes with PBS, the cementoblasts were incubated in alkaline solution for 60 min at room temperature, followed by three washes with PBS to terminate the reaction.

### 2.9. Animal Experiments

Animal experiments were approved by the Biomedical Ethics Committee of Peking University. *In vivo* experiments were performed using 6-week-old male C57BL/6 mice. The mechanical force-induced OTM model was performed using a previously described protocol [23]. After general anesthesia, a nickel–titanium coil spring was placed between the right maxillary first molar and maxillary incisors to provide an almost constant force of approximately 30*g*. The contralateral side was used as a control. After 1 d or 7 d, the springs were removed, and the mice were euthanized by an overdose injection of sodium pentobarbital. The maxilla was carefully removed and fixed in 4% paraformaldehyde solution for 24 h for hematoxylin–eosin (HE) staining and immunostaining.

The local injection model was performed as previously described [24–27]. Mice in the experimental group received injections of 10 mg/ml LPS (from *Escherichia coli*) with 5 *μ*l on each side of the gingival sulcus around the maxillary first to third molars once every 2 days for a total of seven times. The control group was injected with normal saline with the same volume and frequency in the same anatomic regions.

The *in vivo* exosome function experiment used the joint application of local injection on each side of the gingival sulcus around the maxillary first to third molars and caudal vein injection of exosomes once every 2 days for a total of ten times. Mice were randomly divided into three groups, which were subsequently injected with exosomes from M0, M1, or M2, respectively, at the dose of 100 *μ*g per mouse, at 100 *μ*l in volume [28, 29].

### 2.10. HE Staining and Immunohistochemistry Staining

The specimens were cut into 5 *μ*m sections for histologic and immunostaining analysis. HE staining was performed according to the instructions of the HE staining kit (G1120, Solarbio, Beijing, China). For immunohistochemistry staining, sections were rehydrated with a graded ethanol series and incubated with antigen retrieval solution for 10 min at 95°C. To block the activity of endogenous peroxidase, sections were incubated with 3% hydrogen peroxide for 20 min at room temperature. After being blocked with 5% BSA in PBS for 30 min at room temperature, the sections were incubated with primary antibodies. The dilution of primary antibodies depended on the instructions. Appropriate secondary antibodies (ZSGB-Bio, Beijing, China) were applied subsequently. After the production of brown precipitation with a DAB detection kit (Sigma-Aldrich, St. Louis, MO, USA), sections were counterstained with hematoxylin, and images were collected with a microscope (Nikon, Japan).

### 2.11. Data Analysis

Each experiment was repeated independently at least three times. Quantitative data was expressed as means and standard deviation and was analyzed using one-way analysis of variance with IBM SPSS Statistics. A two-tailed *P* < 0.05 was considered to be statistically significant.

## 3. Results

### 3.1. Polarization of Macrophages to M1/M2 Phenotypes

**Figure 1:**
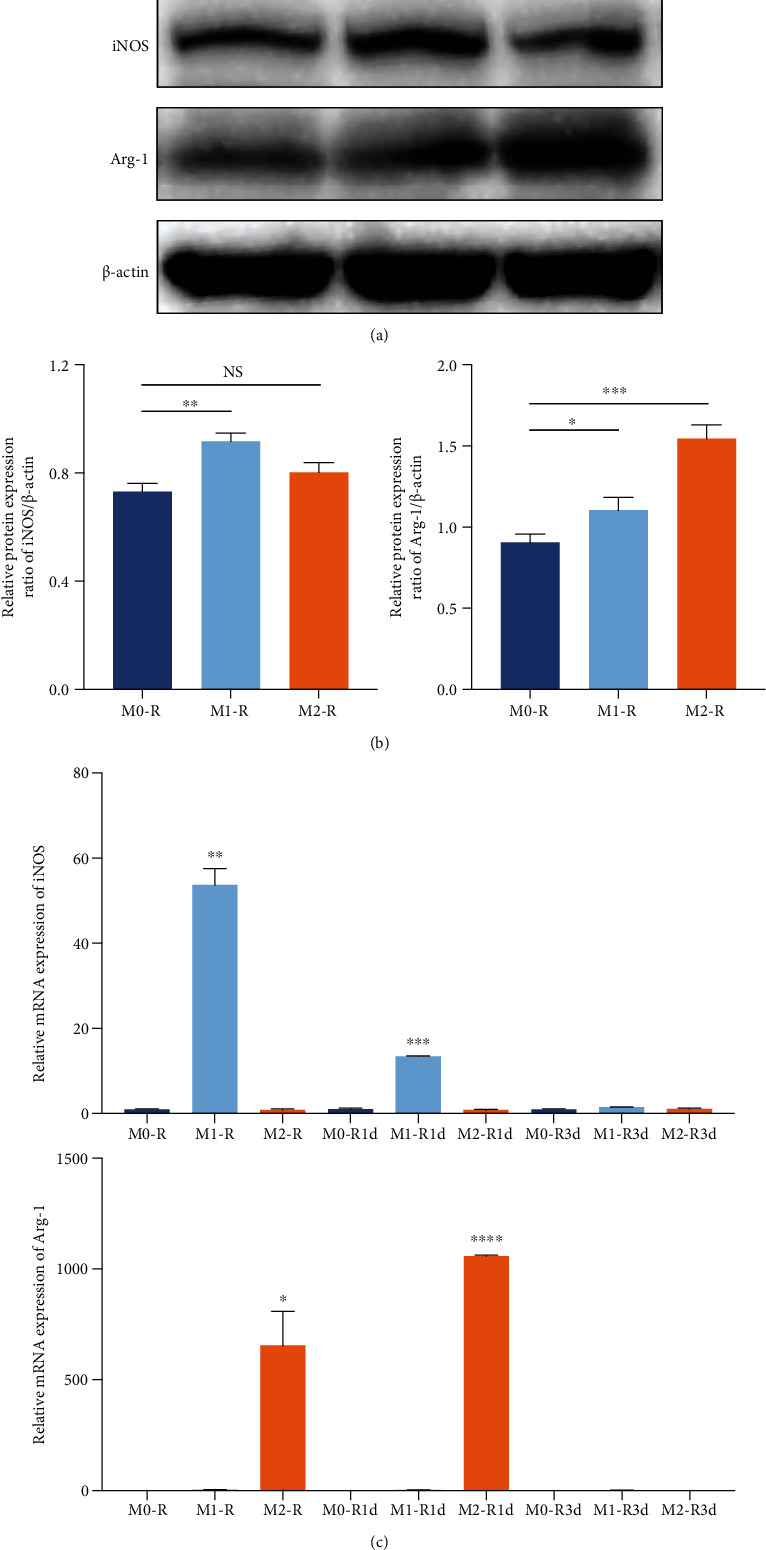
Polarization of macrophages. (a) Representative chemiluminescent images of western blot analysis of iNOS, Arg-1, and *β*-actin in untreated macrophages (referred to as M0-R) and macrophages undergoing M1 or M2 polarization (referred to as M1-R and M2-R). (b) Western blot analysis of iNOS and Arg-1. The gray value was calibrated using *β*-actin. (c) The mRNA expression levels of iNOS and Arg-1 in macrophages untreated or undergoing M1 or M2 polarization immediately (referred to as M0-R, M1-R, and M2-R) or 1 day later (referred to as M0-R1d, M1-R1d, and M2-R1d) or 3 days later (referred to as M0-R3d, M1-R3d, and M2-R3d). ^∗^*P* < 0.05, ^∗∗^*P* < 0.01, ^∗∗∗^*P* < 0.001, and ^∗∗∗∗^*P* < 0.0001.

Macrophages were treated with LPS and IFN-*γ* or IL-4 for 12 h to be polarized into M1 or M2 macrophages, respectively. The M0/M1/M2 proteins were extracted immediately after induction. The protein expression of the M1 polarization marker iNOS was higher in the M1 group than in the M0 and M2 groups. The M2 polarization marker Arg-1 was higher in the M2 group than in the M0 and M1 groups (Figures 1(a) and 1(b)). The mRNAs of M0/M1/M2 were extracted immediately after induction or 1 day or 3 days after induction. The mRNA expression of iNOS was higher in the M1 group compared to the M0 and M2 groups immediately and 1 day after induction, but did not show significant differences 3 days after induction (Figure 1(c)). This suggested that the M1 phenotype polarization effect lasted for 1 day but not until the third day. It was also the fact of M2 phenotype polarization, as the mRNA expression of Arg-1 was higher in the M2 group compared to the M0 and M1 groups immediately and 1 day after induction, but did not show significant differences 3 days after induction (Figure 1(c)). Therefore, in the rest of the experiments, we changed the polarized M1/M2 every day to ensure the polarization effect of M1 and M2.

### 3.2. Macrophages with Different Polarization Phenotypes Influenced Cementoblast Mineralization in Coculture Systems

**Figure 2:**
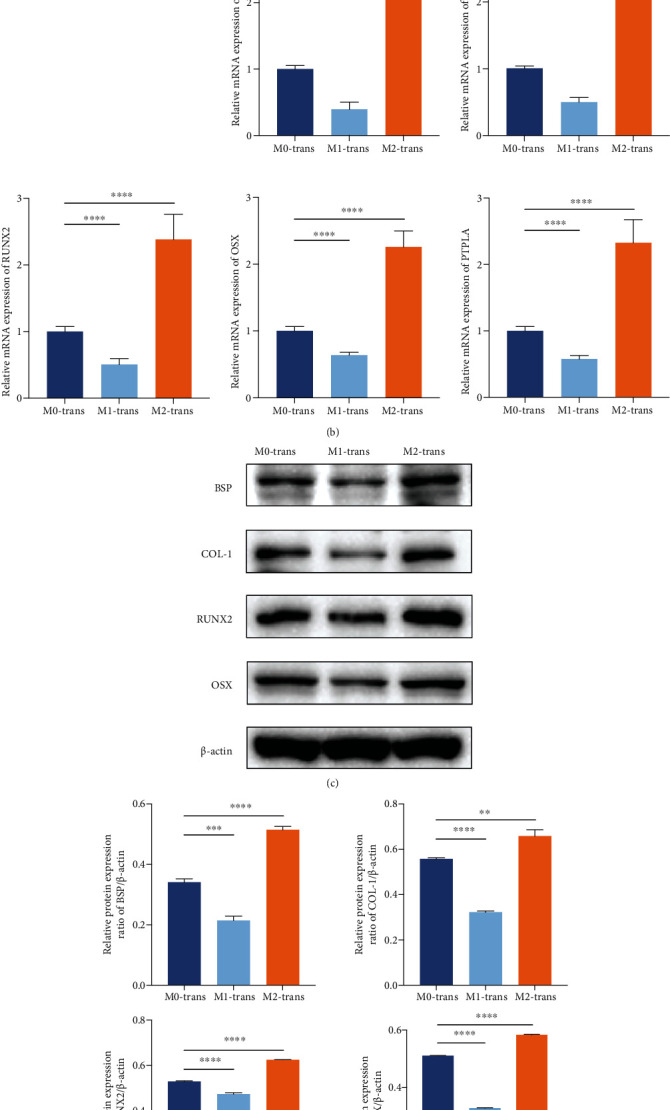
Macrophages with different polarization phenotypes influenced cementoblast mineralization in the transwell coculture system. (a) Diagram of the transwell coculture system of cementoblasts and M0 and M1- and M2-polarized macrophages. (b) The mRNA expression levels of BSP, COL-1, RUNX2, OSX, and PTPLA in cementoblasts after a 3-day coculture with M0, M1, or M2 (referred to as M0-trans, M1-trans, and M2-trans). (c) Representative chemiluminescent images of BSP, COL-1, RUNX2, and OSX western blots from cementoblasts after a 3-day coculture with M0, M1, or M2 (referred to as M0-trans, M1-trans, and M2-trans). (d) Western blot analysis of BSP, COL-1, RUNX2, and OSX. The gray value was calibrated using *β*-actin. ^∗^*P* < 0.05, ^∗∗^*P* < 0.01, ^∗∗∗^*P* < 0.001, and ^∗∗∗∗^*P* < 0.0001.

In the transwell coculture system (Figure 2(a)), the cementoblasts were collected for mRNA and protein extraction after a 3-day coculture. The mRNA expression of mineralization-related genes, including BSP, COL-1, RUNX2, OSX, and PTPLA, was decreased in the M1-polarized macrophage cocultured group and increased in the M2-polarized macrophage cocultured group, compared to the M0 group (Figure 2(b)). The protein expression demonstrated a similar trend (Figures 2(c) and 2(d)). These results suggested that macrophages with different polarization phenotypes, typically referred to as M1- and M2-polarized macrophages, influenced the mineralization of cementoblasts differently compared with M0 and with M1 macrophages attenuating mineralization and M2 macrophages enhancing mineralization.

**Figure 3:**
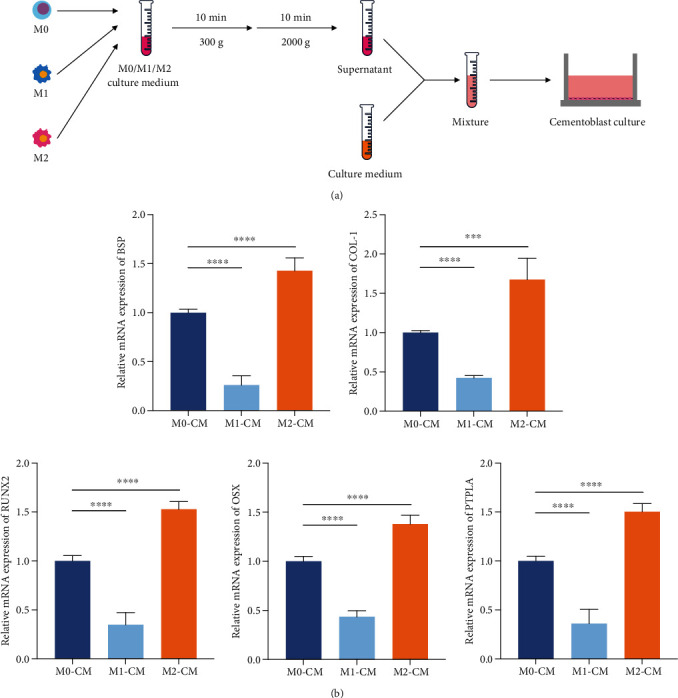
Macrophages with different polarization phenotypes influenced cementoblast mineralization in the conditioned medium-based coculture system. (a) Diagram of the conditioned medium-based coculture system of cementoblasts and M0 and M1- and M2-polarized macrophages. (b) The mRNA expression levels of BSP, COL-1, RUNX2, OSX, and PTPLA in cementoblasts after a 3-day coculture with the supernatant of M0, M1, or M2 (referred to as M0-CM, M1-CM, and M2-CM). ^∗^*P* < 0.05, ^∗∗^*P* < 0.01, ^∗∗∗^*P* < 0.001, and ^∗∗∗∗^*P* < 0.0001.

We further explored the mechanism of how macrophages with different polarization phenotypes affected the mineralization of cementoblasts by performing a conditioned medium-based coculture experiment (Figure 3(a)). The mRNA expression showed a similar trend as in the transwell coculture experiment (Figure 3(b)), which suggested that the influence of macrophages on the mineralization of cementoblasts was mediated by secreted substances from macrophages and was conducted through the culture fluid to the cementoblasts.

### 3.3. Macrophages with Different Polarization Phenotypes Influenced Cementoblast Mineralization through Exosomes

**Figure 4:**
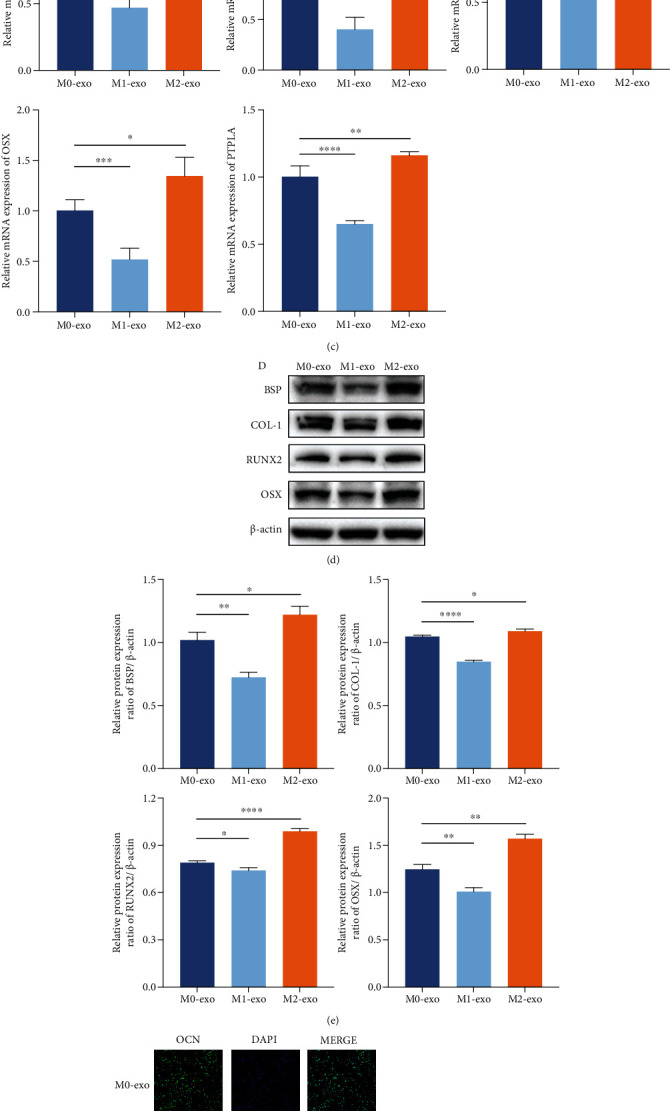
Macrophages with different polarization phenotypes influenced cementoblast mineralization through exosomes. (a) Representative pictures of the exosomes observed by transmission electron microscopy. White arrows indicate the representative exosomes. Scale bars: 100 nm. (b) Representative chemiluminescent images of ALIX, CD63, and GM130 western blots from exosomes. (c) The mRNA expression levels of BSP, COL-1, RUNX2, OSX, and PTPLA in cementoblasts after a 3-day culture with exosomes from M0, M1, or M2 (referred to as M0-exo, M1-exo, and M2-exo). (d) Representative chemiluminescent images of BSP, COL-1, RUNX2, and OSX western blots from cementoblasts after a 3-day culture with exosomes from M0, M1, or M2 (referred to as M0-exo, M1-exo, and M2-exo). (e) Western blot analysis of BSP, COL-1, RUNX2, and OSX. The gray value was calibrated using *β*-actin. ^∗^*P* < 0.05, ^∗∗^*P* < 0.01, ^∗∗∗^*P* < 0.001, and ^∗∗∗∗^*P* < 0.0001. (f) Confocal microscopy of OCN with DAPI counterstaining in cementoblasts after a 3-day culture with exosomes from M0, M1, or M2 (referred to as M0-exo, M1-exo, and M2-exo). Scale bars: 100 *μ*m. (g) ALP staining of cementoblasts after a 5-day culture with exosomes from M0, M1, or M2(referred to as M0-exo, M1-exo, and M2-exo).

Considering that exosomes are important regulators in cellular communication in the cellular supernatant, we extracted exosomes from M0 and M1- and M2-polarized macrophages to examine their function in cementoblast mineralization. The exosomes were verified by western blot analysis and electron microscopy (Figures 4(a) and 4(b)). Exosomes were visualized by transmission electron microscopy, and a vesicular morphology with approximate diameters between 60 and 100 nm was observed. The western blot results showed the presence of the extracellular vesicle marker ALIX and CD63 and the absence of the Golgi marker GM130, suggesting the existence of extracellular vesicles and the absence of Golgi or cell contamination. The effects of adding M0/M1/M2 exosomes to the culture medium of cementoblasts were then assessed. The RT-qPCR and western blot results demonstrated that there was a decrease in the expression of mineralization-related markers in the cementoblast group treated with exosomes from M1-polarized macrophages and an increase in that in the cementoblast group treated with exosomes from M2-polarized macrophages, compared to the cementoblast group treated with exosomes from M0 (Figures 4(c)–4(e)). Immunofluorescence staining also revealed decreased expression of OCN in the cementoblast group treated with exosomes from M1-polarized macrophages and a slight increase in OCN expression in the cementoblast group treated with exosomes from M2-polarized macrophages, compared to the cementoblast group treated with exosomes from M0 (Figure 4(f)). ALP staining of cementoblasts also indicated impaired mineralization of cementoblasts after being treated with exosomes from M1-polarized macrophages and enhanced mineralization of cementoblasts after being treated with exosomes from M2-polarized macrophages, compared to the cementoblast group treated with exosomes from M0 (Figure 4(g)). These results demonstrated that the effect of exosomes from M0/M1/M2 on cementoblast mineralization was in accordance with the effect of M0/M1/M2 on cementoblast mineralization, suggesting that macrophages with different polarization phenotypes influenced cementoblast mineralization, at least partially, through exosomes.

### 3.4. Root Resorption, M1/M2 Accumulation, and Cementoblast Mineralization in the Mechanical Force-Induced OTM Model

**Figure 5:**
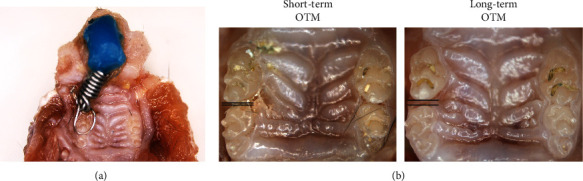
The OTM model and tooth movement induced by mechanical force. (a) Schematic diagram of the OTM model. (b) Tooth movement in the short-term OTM group (1 day) and the long-term OTM group (7 days).

**Figure 6:**
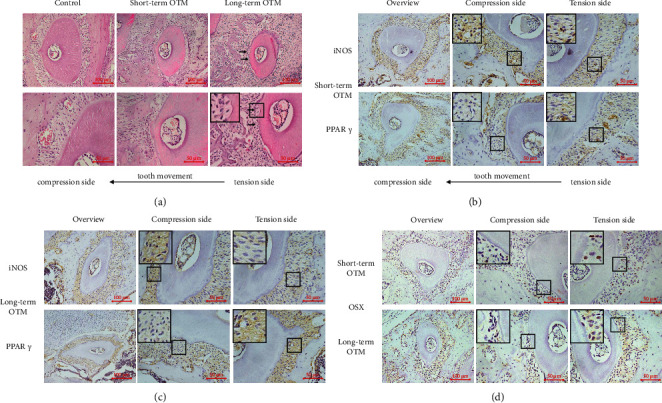
Root resorption, M1/M2 accumulation, and cementoblast mineralization in the mechanical force-induced OTM model. (a) Representative images of HE staining in the roots of the maxillary first molar on the control side and in the short-term OTM (1 d) and long-term OTM (7 d) groups. Black arrows indicate the root resorption zone. Scale bars: 100 *μ*m/50 *μ*m. (b, c) Representative immunohistochemical images of iNOS and PPAR*γ* in the compression side and the tension side of right maxillary first molar roots in the short-term OTM (1 d) and long-term OTM (7 d) groups. Scale bars: 100 *μ*m/50 *μ*m. (d) Representative immunohistochemical images of OSX on the compression side and the tension side of right maxillary first molar roots in the short-term OTM (1 d) and long-term OTM (7 d) groups. Scale bars: 100 *μ*m/50 *μ*m.

To observe root resorption, changes in M1/M2-polarized macrophage accumulation, and mineralization of cementoblasts during OTM, we conducted an *in vivo* study of the mechanical force-induced OTM model (Figure 5). After short-term mechanical force-induced OTM, root resorption could not be seen on either side. After long-term mechanical force-induced OTM, root resorption could be seen on the force side, with no obvious root resorption on the contralateral side (Figure 6(a)). These results confirmed that mechanical force could induce root resorption in the relatively long term, but could not induce obvious root resorption in the short term.

Immunohistochemistry results showed that in the group with short-term mechanical force-induced OTM, the expression of the M1 polarization marker iNOS was slightly higher on the compression side of periodontal tissues than on the tension side, and the expression of the M2 polarization marker PPAR*γ* was slightly lower on the compression side of periodontal tissues than on the tension side (Figure 6(b)). In the group with long-term mechanical force-induced OTM, the expression of the M1 polarization marker iNOS was remarkedly higher on the compression side of periodontal tissues than on the tension side. Conversely, the expression of the M2 polarization marker PPAR*γ* was remarkedly lower on the compression side of periodontal tissues than on the tension side (Figure 6(c)). These data indicated that during the process of mechanical force-induced OTM, M1-polarized macrophages tended to gather on the compression side, while M2-polarized macrophages preferred the tension side. With the extension of force time, the tendency was more obvious, and the difference in the M1/M2 ratio between the compression side and the tension side of periodontal tissues was gradually enlarged.

The mineralization of cementoblasts also showed differences between the compression side and the tension side of cementoblast layers in the process of mechanical force-induced OTM. In the group with short-term mechanical force-induced OTM, the expression of OSX was slightly lower on the compression side of the cementoblast layers than on the tension side. In the group with long-term mechanical force-induced OTM, the expression of OSX was remarkably lower on the compression side of the cementoblast layers than on the tension side (Figure 6(d)). These results suggested that the mineralization of cementoblasts was stronger on the tension side than on the compression side.

### 3.5. Root Resorption, M1/M2 Accumulation, and Cementoblast Mineralization in the Local Injection Model

To further clarify the effects of macrophages on cementoblast mineralization *in vivo* and exclude other influencing factors that may be introduced by OTM, we injected LPS into the gingival sulcus around the maxillary first to third molars of mice to tilt the macrophage polarization toward M1 and used normal saline in the control group.

**Figure 7:**
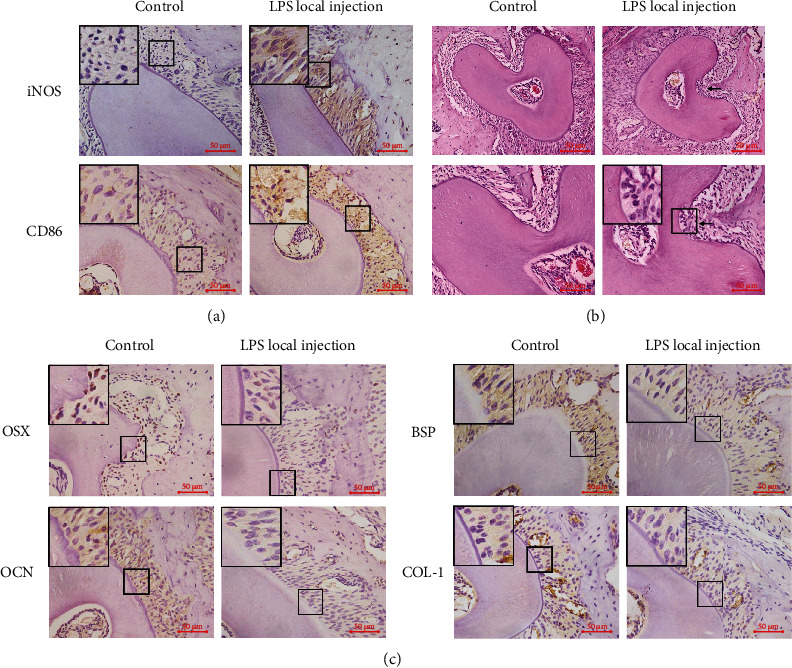
Root resorption, M1/M2 accumulation, and cementoblast mineralization in the local injection model. (a) Representative immunohistochemical images of iNOS and CD86 in the roots of maxillary molars in the control and LPS local injection groups. Scale bars: 50 *μ*m. (b) Representative images of HE staining in the roots of maxillary molars in the control and LPS local injection groups. Black arrows indicate the root resorption zone. Scale bars: 100 *μ*m/50 *μ*m. (c) Representative immunohistochemical images of OSX, OCN, BSP, and COL-1 in the roots of maxillary molars in the control and LPS local injection groups. Scale bars: 50 *μ*m.

Immunohistochemistry results demonstrated that the expression of iNOS and CD86 in the periodontal ligament was increased in the group injected with LPS, compared with the control group, suggesting an enhanced accumulation of M1-polarized macrophages around the roots (Figure 7(a)). In the group injected with LPS, irregularly shaped infiltrated absorption pits could be seen on the roots, while in the control group, there were no obvious signs of root resorption (Figure 7(b)). These results suggested that LPS caused the accumulation of M1 in the periodontal ligament and an increase in the M1/M2 local ratio, as well as the occurrence of tooth root resorption, implying that the M1/M2 ratio was related to the occurrence of root resorption.

Immunohistochemistry results also showed that the expression of OSX, OCN, BSP, and COL-1 was decreased in the periodontal ligament and the layer of cementoblasts in the group injected with LPS, compared with the control group (Figure 7(c)). These results suggested that LPS caused an attenuation of cementoblast mineralization, suggesting a possible relationship between the increase in the M1/M2 ratio, decreased cementoblast mineralization, and the occurrence of root resorption.

### 3.6. Exosomes from Macrophages with Different Polarization Phenotypes Influenced Cementoblast Mineralization In Vivo

**Figure 8:**
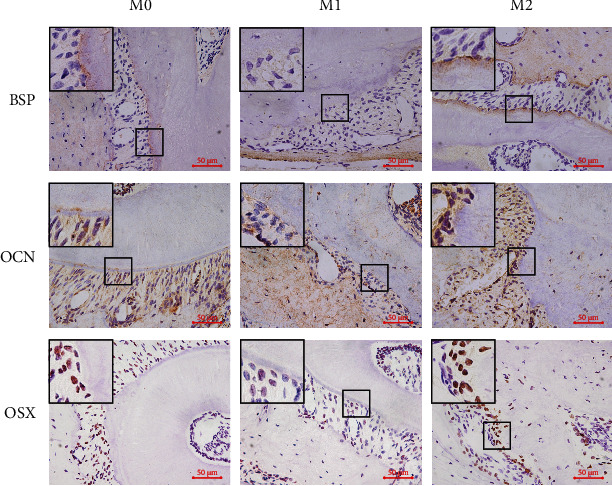
Exosomes from macrophages with different polarization phenotypes influenced cementoblast mineralization *in vivo*. Representative immunohistochemical images of BSP, OCN, and OSX in the roots of maxillary molars in the groups of mice injected with exosomes from M0, M1, or M2. Scale bars: 50 *μ*m.

After the joint application of local injection and caudal vein injection of exosomes, immunohistochemical staining results showed that the expression of BSP, OCN, and OSX was lower in the group injected with exosomes from M1-polarized macrophages, while it was higher in the group injected with exosomes from M2-polarized macrophages, compared to the group injected with exosomes from M0. These results suggested that compared with the exosomes from M0, the exosomes from M1-polarized macrophages would impair the mineralization of cementoblasts *in vivo*, while the exosomes from M2-polarized macrophages could enhance the mineralization of cementoblasts *in vivo* (Figure 8).

## 4. Discussion

Cementoblast is the essential cell type responsible for the maintenance and repair of the cementum, which is closely correlated with the process and repair of root resorption [3]. In addition to its primary role in synthesizing the components of cementum, Nemoto et al. reported that cementoblasts expressed functional Toll-like receptors, which were involved in the alteration of gene expression associated with cementum formation and in the upregulation of osteoclastogenesis-associated molecules, suggesting the additional role of cementoblasts in regulating the metabolism of cementum and bone [8]. In line with this, Huynh et al. also showed that cementoblasts had the capacity to induce osteoclastogenesis, which was strongly promoted by IL-1*β*^7^.

Macrophages also participate in the process of external root resorption, as the removal of necrotic and hyalinized tissues by macrophages is an important cause of the external root resorption [3]. When doing so, macrophages stimulate the osteoclastic cementum destruction, which gives rise to external root resorption [30]. However, macrophages do not unilaterally promote the occurrence of external root resorption. M2-polarized macrophages have been reported to enhance the cementoblastic differentiation of periodontal ligament stem cells via the Akt and JNK pathways [13]. The markers of macrophages have also been shown to diversely affect cementoblast mineralization. The M1 polarization markers IL-1*β*, IL-6, and TNF-*α* were observed to attenuate the mineralization of cementoblasts, while the M2 polarization marker PPAR*γ* was suggested to be a promoter of cementoblast mineralization [14–17]. In the present study, we examined the effect of macrophages with different polarization phenotypes on the mineralization of cementoblasts. Our results indicated that M1-polarized macrophages attenuated cementoblast mineralization, while M2-polarized macrophages facilitated cementoblast mineralization. These effects were, at least partially, exerted by exosomes.

Our findings agreed with the previous study by He et al. showing that root resorption was exacerbated by an increased M1/M2 ratio but was partially rescued by a decreased M1/M2 ratio [12]. We observed that during OTM, the expression of the M1 polarization marker iNOS was higher on the compression side of periodontal tissues than on the tension side, while the expression of the M2 polarization marker PPAR*γ* was higher on the tension side of periodontal tissues than on the compression side, suggesting a relatively higher M1/M2 ratio on the compression side and a relatively lower M1/M2 ratio on the tension side. Together with the external root resorption pit observed on the compression side of the roots, our data supported the finding by He et al. For the reason that macrophages with different polarization phenotypes showed different effects on root resorption, some researchers presented that activated M1-polarized macrophages could induce the recruitment and activation of osteoclasts with the elevated production of caspase-1 and IL-1*β*, which eventually lead to the development of root resorption [31]. In this study, we observed that the mineralization of cementoblasts tended to be attenuated when the M1/M2 ratio increased, resulting in augmented root resorption, while the mineralization of cementoblasts tended to be enhanced when the M1/M2 ratio decreased, resulting in impaired root resorption. Such findings might provide with another possible explanation.

To further clarify the relationship between the M1/M2 ratio and cementoblast mineralization *in vivo*, we actively enhanced the M1 polarization of macrophages by LPS local injection. LPS local injection is a common model for the creation of a proinflammatory environment in the periodontal tissue confirmed by many previous literatures, which also demonstrated that the expression of the M1 polarization markers IL-1*β*, TNF-*α*, and IL-6 was increased during LPS injection, while the M2 polarization marker IL-10 showed no significant change [24–27]. Moreover, Umezu et al. found that in the LPS local injection model, the osteoclast number revealed a dose-dependent increase with injections of 5, 50, or 500 *μ*g/0.05 ml LPS, and with the increase in LPS injection times, the osteoclast count increased progressively, together with the size of osteoclasts and number of nuclei [25]. Therefore, we utilized their protocol parameters in this study, including injection volume, concentration, frequency, and method of configuration. The immunohistochemistry results showed that the expression of iNOS and CD86 in the periodontal ligament was increased in the group injected with LPS compared with the control group, suggesting the enhanced accumulation of M1-polarized macrophages around the roots.

Xiong et al. reported that M2-polarized-macrophage-derived exosomes had the capacity to induce osteogenic differentiation of bone mesenchymal stem cells [32]. It was also observed that exosomes from M0 and M2-polarized macrophages promoted bone repair or regeneration, while exosomes from M1-polarized macrophages inhibited bone repair, suggesting that macrophages with different polarization phenotypes could influence bone metabolism via exosomes [11]. Therefore, we also investigated whether the effect of macrophages on cementoblasts was mediated by exosomes. We collected exosomes from M0 and M1- and M2-polarized macrophages and added them to the culture medium of cementoblasts. It turned out that exosomes from M1 attenuated the mineralization of cementoblasts compared with exosomes from M0, while exosomes from M2 showed the opposite effect, which is consistent with the coculture effects of macrophages on cementoblast mineralization and the effects of macrophage supernatant on cementoblast mineralization. The *in vivo* experiment also came to a similar result, as the expression of BSP, OCN, and OSX was lower in the group injected with exosomes from M1-polarized macrophages, while higher in the group injected with exosomes from M2-polarized macrophages, compared to the group injected with exosomes from M0. These results suggested that the effects of macrophages on cementoblast mineralization were, at least partially, mediated by exosomes.

When it came to the clinical significance, our study presented a perspective of the intercellular interactions in external root resorption that may be helpful for a better understanding of the role that these intercellular interactions play during this process. By studying the relationship between macrophages and cementoblast mineralization, novel methods may be developed to maintain the balance of macrophages between different polarization phenotypes or to facilitate the M2 polarization to minimize the extent of root resorption and to promote the transition from resorption to repair. New regulatory factors may also be found between macrophages and cementoblasts in future studies, which may serve as a breakthrough in the exploration of controlling root resorption.

## Figures and Tables

**Table 1:** mRNA primer sequences used in this study.

Gene	Forward sequence (5′→3′)	Reverse sequence (5′→3′)
GAPDH	ACCACAGTCCATGCCATCAC	TCCACCACCCTGTTGCTGTA
BSP	GAGACGGCGATAGTTCC	AGTGCCGCTAACTCAA
OSX	TTGAAAAAGGAGTTGGTGGC	TGCTGGTTCTGTAAGTTGGG
RUNX2	CCTGAACTCTGCACCAAGTCCT	TCATCTGGCTCAGATAGGAGGG
COL-1	GCAACATTGGATTCCCTGGACC	GTTCACCCTTTTCTCCCTTGCC
PTPLA	AGCCCAGGTATAGGAAGAATGT	CCGCATAACTAACCCAATAGCG
iNOS	CAGCACAGGAAATGTTTCAGC	TAGCCAGCGTACCGGATGA
Arg-1	CTCCAAGCCAAAGTCCTTAGAG	AGGAGCTGTCATTAGGGACATC

## Data Availability

The data used to support the findings of this study are included within the article.
